# Phylogenetic Analysis and Epidemic History of Hepatitis C Virus Genotype 2 in Tunisia, North Africa

**DOI:** 10.1371/journal.pone.0153761

**Published:** 2016-04-21

**Authors:** Mouna Rajhi, Kais Ghedira, Anissa Chouikha, Ahlem Djebbi, Imed Cheikh, Ahlem Ben Yahia, Amel Sadraoui, Walid Hammami, Msaddek Azouz, Nabil Ben Mami, Henda Triki

**Affiliations:** 1 Pasteur Institute, Tunis, Tunisia; Laboratory of Clinical Virology, WHO Regional Reference Laboratory on Poliomyelitis and Measles, Tunis, Tunisia; 2 Pasteur Institute, Tunis, Tunisia; Laboratory of Bioinformatics, Mathematics and Statistics, Tunis, Tunisia; 3 Department of Gastroenterology, La Rabta Hospital, Tunis, Tunisia; 4 Department of Gastroenterology, Regional Hospital of Bizerte, Bizerte, Tunisia; 5 Department of Gastroenterology, Regional Hospital of Nabeul, Nabeul, Tunisia; 6 University of Tunis El Manar, Tunis, 1036, Tunisia; 7 University of Carthage, Faculty of Sciences, Bizerte, Tunisia; University of Cincinnati College of Medicine, UNITED STATES

## Abstract

HCV genotype 2 (HCV-2) has a worldwide distribution with prevalence rates that vary from country to country. High genetic diversity and long-term endemicity were suggested in West African countries. A global dispersal of HCV-2 would have occurred during the 20^th^ century, especially in European countries. In Tunisia, genotype 2 was the second prevalent genotype after genotype 1 and most isolates belong to subtypes 2c and 2k. In this study, phylogenetic analyses based on the NS5B genomic sequences of 113 Tunisian HCV isolates from subtypes 2c and 2k were carried out. A Bayesian coalescent-based framework was used to estimate the origin and the spread of these subtypes circulating in Tunisia. Phylogenetic analyses of HCV-2c sequences suggest the absence of country-specific or time-specific variants. In contrast, the phylogenetic grouping of HCV-2k sequences shows the existence of two major genetic clusters that may represent two distinct circulating variants. Coalescent analysis indicated a most recent common ancestor (tMRCA) of Tunisian HCV-2c around 1886 (1869–1902) before the introduction of HCV-2k in 1901 (1867–1931). Our findings suggest that the introduction of HCV-2c in Tunisia is possibly a result of population movements between Tunisia and European population following the French colonization.

## Introduction

Hepatitis C virus (HCV) is one of the most important causes of chronic liver disease worldwide. Since its discovery in 1989 [1], several isolates from various regions of the world were sequenced and a high degree of genomic heterogeneity was observed. Thus, HCV strains are currently classified into seven genotypes (1 to 7) and several subtypes [2]. Genotype identification is clinically important to predict response to antiviral therapy, as some genotypes are more resistant to treatment than others; it is now routinely performed before treatment and serves as a guideline to determine the duration of the therapy [3,4]. Genotyping is also useful for molecular epidemiology studies. The geographical distribution of HCV genotypes and subtypes varies significantly from a region to another in the world [5]. Genotypes 1, 2, and 3 have a worldwide distribution, while genotypes 4, 5 and 6 are mainly encountered in Egypt, Middle East and Central Africa, Southern Africa and Asia, respectively [6–9].

HCV genotype 2 (HCV-2) is worldwide distributed but is reported to be more frequent in West African countries and Martinique [10–13]. It is one of the HCV genotypes showing the widest genomic diversity. Twenty-one subtypes (designated a to u) are presently assigned in the HCV sequence database [14]. Subtypes 2a, 2b and 2c are ubiquitous while others subtypes are mainly found in specific regions [15].

In Tunisia, Genotype 1 is the most prevalent, representing 70 to 80% and Genotype 2 comes in the second position with approximately 10% of circulating isolates [16–18]. In a recently published study, we showed that most of HCV-2 isolates belong to subtype 2c with few co-circulating isolates from other subtypes, especially 2k [19]. The aim of the present study was to analyse the genetic diversity and to reconstruct the genetic history of the most prevalent HCV-2 subtypes. The NS5B sequences of 113 isolates from subtypes 2c and 2k, collected over a 10-years period in Tunisia, were analyzed by phylogeny and a Bayesian coalescent investigation was carried out to estimate the origin and the evolution of these two subtypes.

## Material and Methods

The samples used in the present study were obtained as part of the routine diagnostic activity of the Laboratory of Clinical Virology in Pasteur institute of Tunis and this did not need an ethical approval. HCV Viral load and genotyping were performed in response to the request of the physicians. Patient records/information was anonymized and de-identified prior to inclusion in the study.

### Study population

The study included serum samples from 113 Tunisian patients who were detected HCV RNA-positive and the isolated viruses belonged to HCV subtype 2c (N = 102) or HCV subtype 2k (N = 11). The serum samples were collected over a 10-years period, from 2003 to 2012.

During the study period, 1430 serum samples were processed for genotyping, 1171 (81%), 135(9%), 63(4%) and 29(2%) belonged to genotypes 1, 2, 3 and 4, respectively. Subtyping of the HCV-2 isolates revealed that most of them belonged to subtype 2c (75%), followed by Subtype 2k (8%). All the isolates belonging to these two subtypes were included in the present study. The study population included 86 women and 27 men. All of them were Tunisians, they originated from all regions of Tunisia and were aged 24 to 70 years with a mean age of 51 years.

### PCR amplification and sequencing

RNA extraction was performed from 140μl of serum using the QIAmp viral RNA mini Kit (QIAGEN, Hilden-Germany) and the extracted RNA was then processed for reverse transcription (RT) and PCR amplification. The RT reaction mixture included 10μl of viral RNA, 10pmol of random hexamers, 1μl of 40U RNasin (Promega), 4μl of 5X transcriptase buffer, 2μl of 0,1M DTT, 1μl of dNTP mix (20mM) and 200U of the super script II Reverse Transcriptase (Invirogen); in a final volume of 20μl. The RT reaction mixture was incubated at 42°C for 45min then heat/inactivated at 94°C for 5min. A 364-nucleotide fragment in the NS5B region (positions 8267 to 8630) was amplified by nested PCR using two pairs of primers specific to relatively conserved sequences in the NS5B genomic region: **Outer primers:** 1s (Forward (8256–8275): 5’-TATGAYACCCGCTGYTTTGAC-3’), 2as (Reverse (8622–8641): 5’-GARTACCTRGTCATAGCCTC-3’) and **inner primers** 3as (Forward (8267–8287): 5’-CTGYTTTGACTCMACRGTCAC-3’), 4as (Reverse (8611–8630): 5’- ATAGCCTCCGTGAAGRCTC-3’). The first round of PCR was performed with 10μl cDNA in a total reaction mixture of 50μl containing 5μl 10X polymerase buffer, 3μl MgCl_2_, 5U recombinant-Taq polymerase and 10pmol each forward and reverse outer primers. The same conditions were used in the second round of PCR using5μl of first round PCR product and 10pmol of each inner primer. The cycling profile was identical for the two rounds and consisted in a pre-denaturation at 95°C for 5 min followed by 30 cycles at 94°C for 30s, 60°C for 30s and 72°C for 1min. A final extension was performed at 72°C for 7min. The PCR products were separated and visualized on a 1% agarose gel. The PCR products were excised from the gel and purified with the QIAquick Gel Extraction kit (QIAGEN). The purified templates DNA were sequenced in both directions using the Big-Dye terminator ready reaction cycle sequencing Kit (Applied Biosystems) and the same inner primers on an ABI Prism 3130 automated sequencer (Applied Biosystems).

### Phylogenetic analyses

Ancestral relationships among HCV isolates were inferred by reconstructing phylogenetic trees of Tunisian NS5B sequences, together with the HCV-2c and HCV-2k sequences available in GenBank database.

Phylogenetic analyses were performed using the Maximum composite likelihood methods included in the MEGA package (Version 5.1). Although the Tunisian sequences were more than 300-nucleotide-long, the phylogenetic analyses were performed on a fragment of 222 nucleotides of the NS5B region in the aim to include the maximum of sequences from other countries. The reliability of the phylogenetic constructions was estimated by bootstrap analysis with 1000 pseudo replicate data sets. Sixty-eight out of the 113 Tunisian sequences included in the present study were previously published in GenBank [19] whereas 45 sequences are new and reported for the first time in this study; they were submitted to GenBank under the accession numbers KJ206290 to KJ206333 and KJ206335. In addition, ninety-two HCV field sequences, published in GenBank and isolated from different countries in the world were included in these phylogenetic analyses: 53 isolates from Subtype 2c and 39 isolates from subtype 2k. The selected sequences represented the widest temporal and geographical range including 18 different countries and an isolation year ranging from 1993 to 2013.

### Calculation of the genetic distances

Estimates of evolutionary divergence between the sequences were conducted using the maximum likelihood method included in the MEGA 5.1 software.

### Coalescent analyses

The epidemic history of HCV subtypes 2c and 2k was investigated with a coalescent-based strategy. Briefly, HCV evolutionary history was inferred by Bayesian Markov Chain Monte Carlo (MCMC) analysis implemented in BEAST software version 1.8.1 (http://beast.bio.ed.ac.uk) [20]. For this analysis, five population dynamics models were used: Bayesian, constant, exponential, expansion and logistic under the strict and relaxed clocks. In all cases, the Hasegawa-Kishino-Yano (HKY) nucleotide-substitution model [21] was used under the strict then relaxed molecular clock. All BEAST chains were run for 3x10^8^ generations for HCV- 2c and for 1.5x10^8^ generations for HCV-2k in order to achieve an Effective Sample Size (ESS)>200 for the 2 subtypes. Since the time interval covered by the sequences of HCV-2c and HCV-2k for the NS5B region with known sampling date is about 10 years, the substitution rate parameter could not be reliably estimated, and thus an external substitution rate was used. A substitution rate of 5x10^-4^ substitutions per site per year (s/s/y) was used according to previous estimation [22] for the NS5B region. Best fitting models were selected by calculation of a Bayes Factor (BF) [23], using Marginal Likelihoods Estimation (MLE) specifically path sampling (PS) [24,25] and stepping-stone (SS) [26] sampling implemented in Beast 1.8.1.

The effective number of infections through time was estimated by using the Bayesian skyline plot (BSP) approach [27] under the strict and relaxed molecular clock models. All BEAST output log files were analyzed with TRACER v1.6 program (Available from http://tree.bio.ed.ac.uk/software/tracer/). The present analysis was run on two different datasets of NS5B sequences: subtype 2c (n = 102, 307nt, 8302 to 8608), subtype 2k (n = 11, 307nt, 8302 to 8608).

## Results

### Phylogenetic analyses

#### Subtype 2c

**Fig 1:**
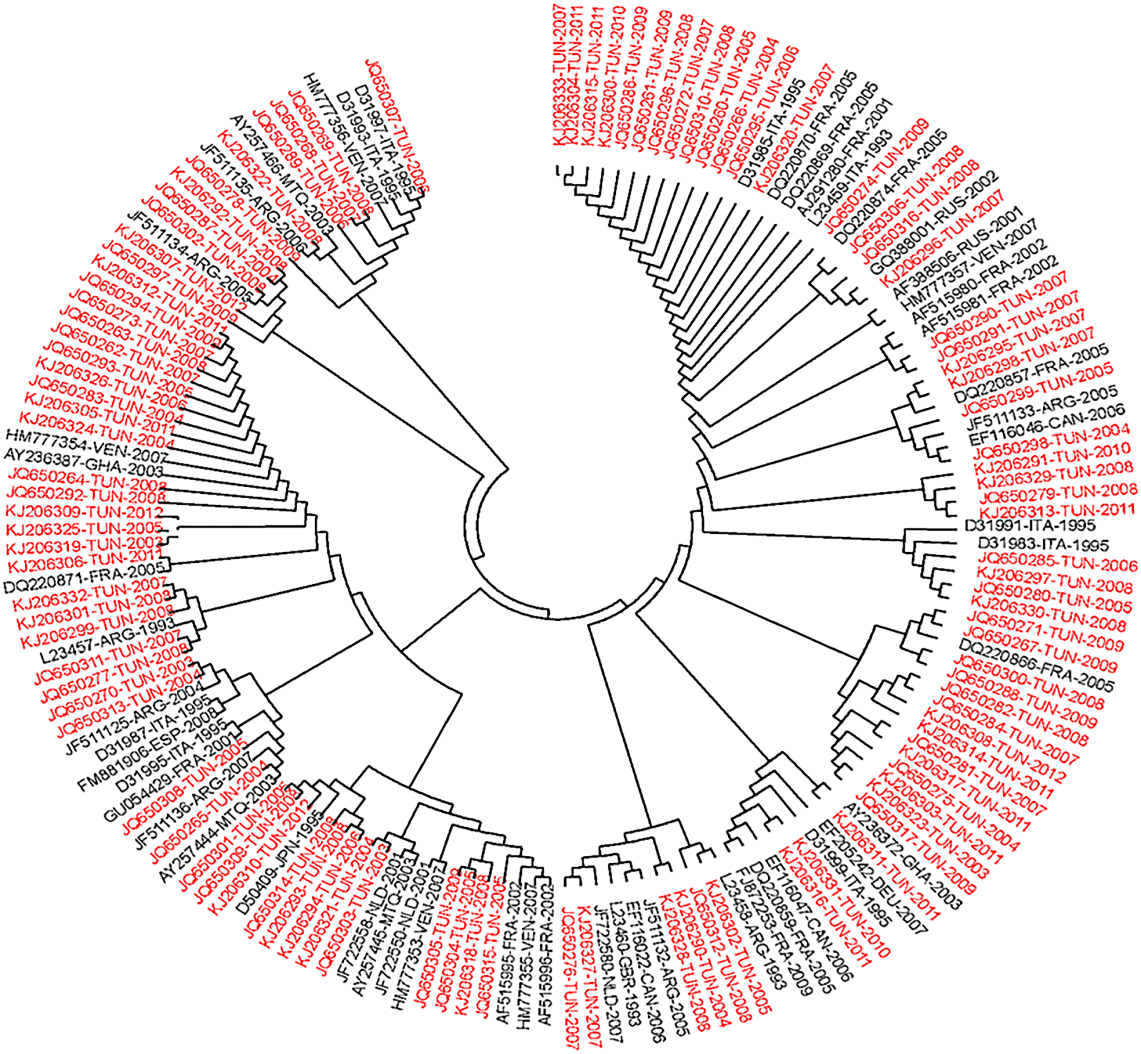
Phylogenetic analysis of isolates from subtype 2c. Phylogenetic analyses were performed using the Maximum Composite Likelihood method included in the MEGA package (version 5.1). The reliability of the phylogenetic constructions was estimated by bootstrap analysis with 1000 pseudo replicate data sets. The tree is based on the analysis of a 222-nucleotide-long fragment in the NS5B region (nucleotides 8316 to 8537 according to the H77-1a prototype strain, AF009606). It includes the 102 studied Tunisian sequences (in red) and 53 HCV-2c field sequences from other countries (in black). All the sequences were indicated by their Accession Numbers followed by the country code and the collection date. The country codes according to the standard abbreviation are: France (FRA), Martinique (MTQ), Canada (CAN), Italy (ITA), Venezuela (VEN), Ghana (GHA), Russia (RUS), Netherlands (NLD), Spain (ESP), Japan (JPN), Germany (DEU), Argentina (ARG) and United Kingdom (GBR).

The phylogenetic tree in Fig 1 contains 102 Tunisian sequences that overlapped a fragment of 222 nucleotides in the NS5B genomic region; the corresponding virus isolates were already classified within subtype 2c. The tree also includes 53 field sequences published in GenBank and originating from 13 different countries: France (N = 14), Martinique (N = 3), Canada (N = 3), Italy (N = 9), Venezuela (N = 5), Ghana (N = 2), Russia (N = 2), Netherlands (N = 3), Spain (N = 1), Japan (N = 1), Germany (N = 1), Argentina (N = 8) and United Kingdom (N = 1). No consistent cluster supported by a high bootstrap value could be identified and the Tunisian sequences were randomly distributed throughout the phylogenetic tree, among all subtype2c sequences that formed a monophyletic group. The divergence rates between all the 2c sequences, in the studied region, varied from **0%** to **10.7%** with an overall mean distance of **2.6%**.

#### Subtype 2k

**Fig 2:**
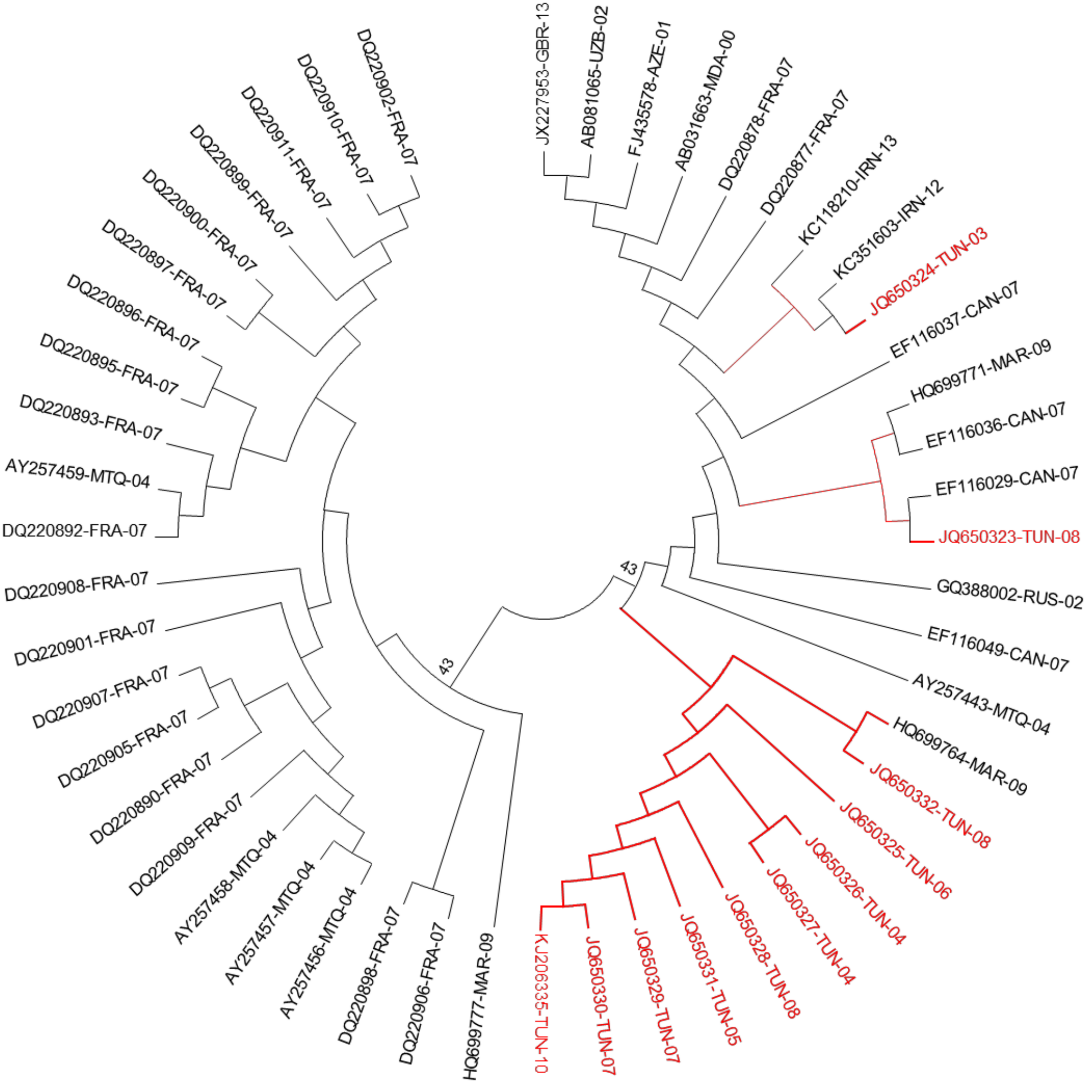
Phylogenetic analysis of isolates from Subtype 2k. Phylogenetic analyses were performed using the Maximum Composite Likelihood method included in the MEGA package (version 5.1). The reliability of the phylogenetic constructions was estimated by bootstrap analysis with 1000 pseudo replicate data sets. The tree is based on the analysis of a 222-nucleotide long fragment in the NS5B region (nucleotides 8346 to 8568 according to the H77-1a prototype strain, AF009606). It includes the 11 studied Tunisian sequences (in red) and 39 HCV-2k field sequences from other countries (in black). All the sequences were indicated by their Accession Numbers followed by the country code and the collection date. The country codes according to the standard abbreviation are: France (FRA), Martinique (MTQ), Canada (CAN), Russia (RUS), United Kingdom (GBR), Uzbekistan (UZB), Azerbaijan (AZE), Morocco (MAR), Iran (IRN) and Moldova (MDA).

The phylogenetic tree in Fig 2 includes 11 Tunisian sequences previously classified within subtype 2k and 39 field sequences of the same subtype reported from 10 different countries: France (N = 20), Canada(N = 4), Russia(N = 1), Martinique(N = 5), Moldova(N = 1), Uzbekistan(N = 1), Azerbaijan(N = 1), Morocco(N = 3), United kingdom(N = 1) and Iran(N = 2). All the sequences segregated into two clusters: Cluster1 comprised most of French sequences (18 out of 20), four out of the five sequences from Martinique and one out of the three sequences from Morocco. Cluster2 had wider geographic distribution and contained all the Tunisian sequences together with sequences from all the ten other countries. The divergence rates within subtype 2k, in the studied NS5B region, ranged from **0.6** to **13.6%** with an overall mean distance of **6.9%**.

### Epidemic history of HCV-2c and HCV-2k circulating in Tunisia

#### Bayesian Skyline Plots

**Fig 3:**
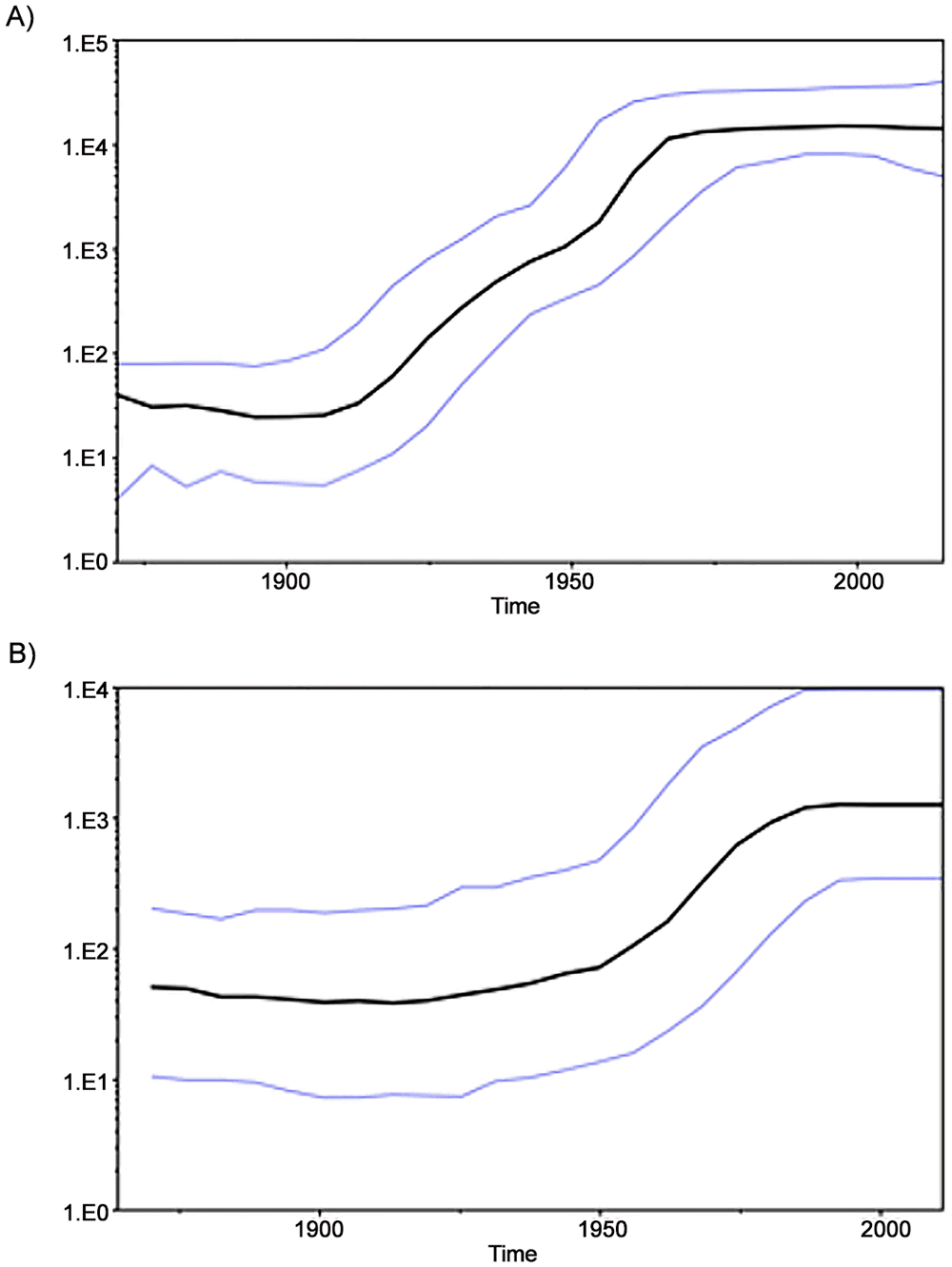
Bayesian Skyline Plots for Demographic Reconstruction using NS5B sequences. A) subtype 2c, B) subtype 2k; X-axis: Date in Years; Y-axis: Estimated effective number of infections; Bold Line: Mean Effective Number of viral population. Upper and lower Lines: Upper and Lower HPD95% (High Population Density) of Effective Number of viral population.

Bayesian coalescence analysis was performed on all the NS5B sequences from Tunisia, separately for the subtype 2c dataset (102 sequences) and the subtype 2k dataset (11 sequences). For each demographic and molecular clock models, chain lengths of 300 million for HCV-2c and of 150 million for HCV-2k were used and sampled every 30000 and 15000 states, respectively. For HCV-2c, the best-fitted model was the exponential growth with relaxed molecular clock. For HCV- 2k, the best-fitted model was the logistic growth with strict molecular clock. The data was analysed using the Bayesian Skyline Plot (BSP) that depicts the estimated change in the effective number of infected individuals over time for each subtype. -2c and HCV-2k (Fig 3a and 3b) exhibited a similar epidemic history, characterized by three phases of epidemic population growth including: an initial period of relatively constant population size, followed by a well-defined phase of exponential growth ranging from 1912 to 1970 for HCV-2c and from 1940 until 1985 for HCV-2k; the final stabilization phase starts around 1970 for subtype 2c and around 1985 for subtype 2k.

#### Most Recent Common Ancestor (tMRCA)

The most recent common ancestor (tMRCA) was evaluated by using a fixed substitution rate for the NS5B region of 5.10^−4^ substitutions per site and per year according to a previous study [22]. The tMRCA of subtype 2c was evaluated around 1886 (1869–1902), prior to the introduction of subtype 2k estimated in 1901 (1867–1931) (Table 1).

**Table 1:** _t_MRCA estimations using the NS5B region for the HCV- 2c and HCV-2k in Tunisia.

HCV subtype	Number of isolates	Substitution rate	MRCA (HPD^1^ 95%) (year)
2c	102	5.10^−4^ s/site/year	1886 (1869–1902)
2k	11	5.10^−4^ s/site/year	1901 (1867–1931)

(^1^: HPD: Highest Posterior Density).

## Discussion

HCV-2 has a worldwide distribution with prevalence rates that vary from country to country. Previous studies suggested that it originated from Africa where it would have endemically persisted for 500 to 600 years [28–31]. A global dispersal of HCV-2 would have occurred during the 20^th^ century and in some countries such as Venezuela, a reduction in the circulation of HCV-1 was observed in the last decade with an increase of circulation of genotype 2 [32]. Recently, the genetic diversity of HCV-2 and its epidemic history have been assessed in several countries, mainly from Europe and Latin America [33–36] but such investigations remain very scarce for HCV-2 isolates circulating in North African countries.

Among the different HCV-2 subtypes, HCV-2c is reported to be ubiquitous worldwide. It was identified in several countries, contrary to the other subtypes, which seem to have more restricted geographical distribution [15]. The frequency of HCV-2c varies from one country to another. Although not very common in Brazil, Canada, Chile, Mexico, or the USA [37,38], HCV-2c is the second most frequently isolated after genotype 1 in Argentina [39], Italy [40] and in Tunisia [19]. In the present work, we analyzed the genetic diversity and the phylogenetic relatedness between the NS5B sequences within HCV-2c subtype. These analyses were performed on 102 Tunisian sequences and 53 representative sequences from 13 other countries. The genetic distance found between all HCV-2c sequences did not exceed 10.7%, with an overall mean distance of 2.6%. Several Tunisian strains were identical to each other and to other sequences from France and Italy. Previous studies reported low genetic diversity of HCV-2c and suggested that it is an epidemic subtype [22,41]. Furthermore, all HCV-2c sequences formed a monophyletic group; the Tunisian sequences were intermixed with the other sequences suggesting the absence of country-specific or time-specific variants. Similar results were also reported in previous studies and the authors suggested that the low genetic diversity within HCV-2c sequences from various geographical origins would be a reflection of its quick spread during the 20^th^ century [22,35,41].

In the other hand and contrary to HCV-2c, the phylogenetic analysis of HCV-2k sequences showed different features. The genetic diversity within the subtype was higher and the phylogenetic analysis of the 11 sequences from Tunisia and 39 sequences representative of 10 other countries showed the existence of two major genetic clusters that may represent two distinct circulating variants. All Tunisian isolates belonged to the same variant. Subtype 2k was reported from several countries; France, Canada, Russia, Martinique, Moldova, Uzbekistan, Vietnam, Madagascar, Azerbaijan, Morocco, United Kingdom and Iran. In southwestern France, subtype 2k is the second most frequent subtype of circulating HCV-2 isolates with a prevalence of 22% [34]. However, it remains much less investigated compared to HCV-2c and, to our knowledge, beyond prevalence rates, its molecular epidemiology still insufficiently documented.

Recent works highlighted the role of human migration process in the global and rapid dissemination of HCV genotypes [35,42,43]. Some studies suggested the introduction of HCV-2 in western countries from the African continent probably in the 17th-18th centuries through the slave trade and colonialism [28,33,36, 44]. Studies conducted on the genetic history of HCV genotype 2 mainly concerned subtype 2c and proposed the following dates for the tMRCA: 1791 in France [42], 1871 in Argentina [35], 1889 in Italy [43]. The tMRCA of subtype 2j in Venezuela was evaluated around 1785 [33]. The epidemic history of HCV is rarely studied in North Africa. To our knowledge, the only report in this topic is the one of Brahim et al. [45] who established the genetic history of HCV-1b and HCV-2i in Morocco. In the present work, the epidemic history of HCV-2c and HCV-2k Tunisian strains was estimated with a coalescent approach. The results suggest that the subtype 2c would have been introduced in the country at the end of the 19^th^ century, with an estimated tMRCA date in 1886 (1869–1902) and that HCV-2k would have been introduced in the beginning of the 20^th^ century, around 1901 (1867–1931). Also, the Bayesian Skyline Plot shown in Fig 3 suggests that the 2c infected population increased exponentially between 1912 and 1970 while the 2k infected population experienced exponential growth later, between 1940 and 1985.

Looking on the history of Tunisia, the introduction of the HCV-2c may have been caused by the contact of the Tunisian population with European populations following the French colonization. Between the 19th and the early 20th century, the Italian community in Tunisia represented the largest group of European origin. At the beginning of the 19th century, it included several thousands of political activists and intellectuals from the central and northern regions of Italy, sheltering in the Regency of Tunis. At the end of the 19th century, the economic difficulties and the social crisis in the southern regions of the newly established Kingdom of Italy contributed to a coutinuous increase in the Italian population living in Tunisia with the arrival of migrants mainly from Sicily, Pantelleria, Sardinia and Procida [45]. Tunisia became a French protectorate in 1881 and the French population in Tunisia increased. However, the Italian community continued to grow and represented the main European community in the French protectorate with more than 100,000 Italians in the first years of the 20th century; Sicilians made up 72.5% of the community's population, while 16.3% were from central Italy, 3.8% from Sardinia and only 2.5% from northern Italy [46]. Thereafter, the colonization of Tunisia by French expatriates was followed by the return-back of Italians from Tunisia to Italy. Between 1943 and 1970, the Italian community almost disappeared from Tunisia.

**Fig 4:**
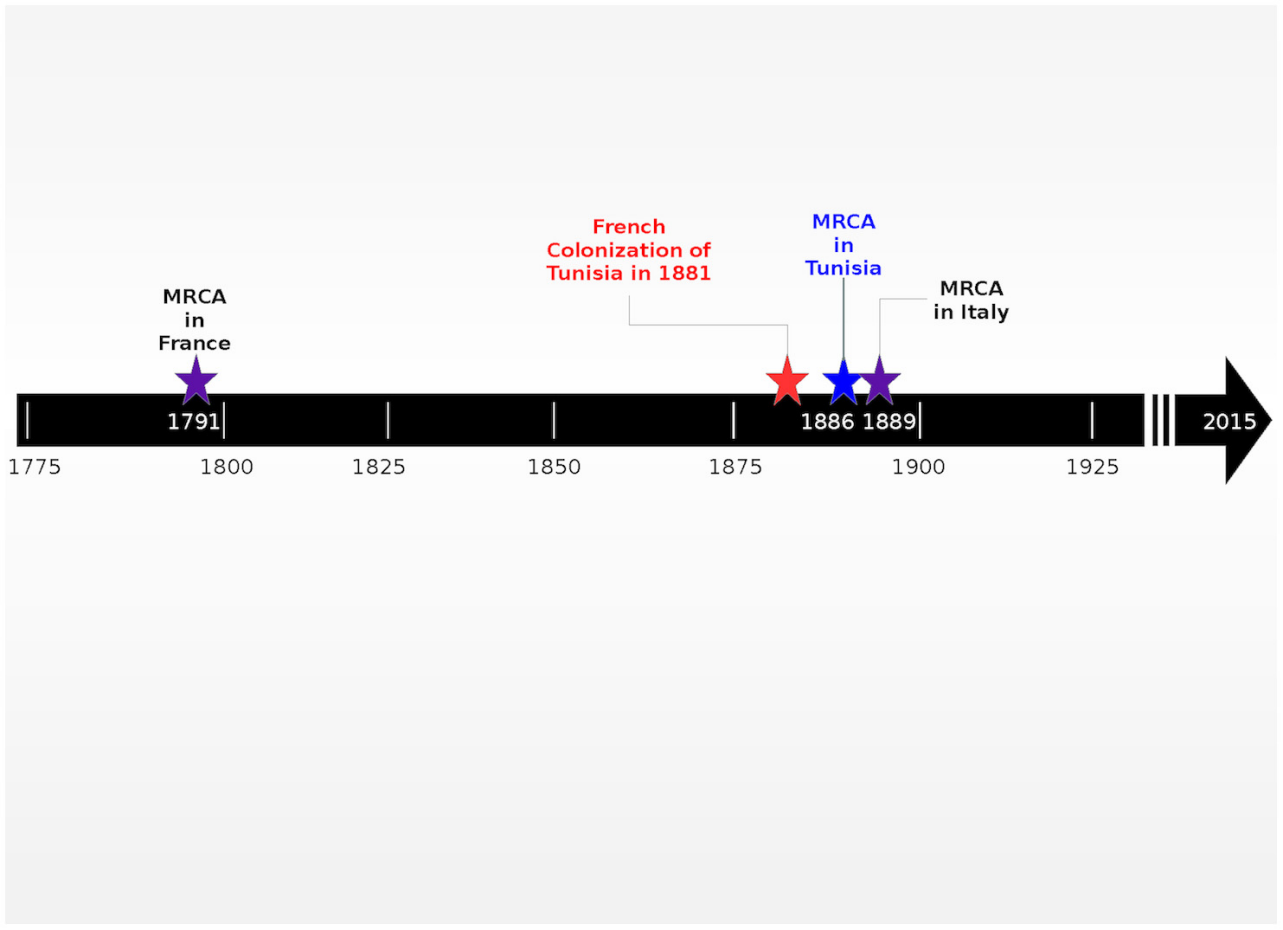
The timeline of HCV subtype 2c introduction in Tunisia.

Thus, if we consider the dates of the HCV-2c MRCA estimated in 1791 in France [42], in 1886 in Tunisia (the present work) and in 1889 in Italy [44], we may conclude that HCV-2c would have been introduced first in France and then in Tunisia and Italy, 90 to 100 years later, possibly as a result of population movements between Tunisia and European Countries after the French colonization (Fig 4). The massive return back of Italians from Tunisia to Italy during the 20^th^ century may have contributed to the high prevalence of HCV-2c especially in southern Italy [40, 43].

## Conclusion

In conclusion, this study combined demographic, historical and viral genetic data to reconstruct the transmission history of HCV-2 in Tunisia. To our knowledge, it is the first to describe the evolutionary history of subtype 2k at the international level and is the first study on the diversity and genetic history of HCV-2c in a country from North Africa. Our results indicate that the epidemiology of HCV-2 in Tunisia was mainly influenced by population movements between countries of the Mediterranean basin especially those from the north bench of the Mediterranean Sea. Through a better understanding of past HCV-2 transmission events, this study helps to better explain the worldwide distribution of HCV-2 and to define risk factors and preventive measures for HCV-related diseases.
